# Epigenetic modifications in follicular cell-derived thyroid cancer: new dimensions in pathogenesis and treatment

**DOI:** 10.3389/fonc.2025.1549477

**Published:** 2025-05-20

**Authors:** Yanmei Han, Bian Wu, Jie Tan, Ruolin Wu, Jingjing Wang, Xiaojing Ren, Yajing Zhang, Zairong Gao, Xiaotian Xia

**Affiliations:** ^1^ Department of Nuclear Medicine, Union Hospital, Tongji Medical College, Huazhong University of Science and Technology, Wuhan, China; ^2^ Hubei Province Key Laboratory of Molecular Imaging, Wuhan, China; ^3^ Key Laboratory of Biological Targeted Therapy, the Ministry of Education, Wuhan, China; ^4^ Cancer Center, Union Hospital, Tongji Medical College, Huazhong University of Science and Technology, Wuhan, Hubei, China; ^5^ Department of Breast and Thyroid Surgery, Union Hospital, Tongji Medical College, Huazhong University of Science and Technology, Wuhan, Hubei, China

**Keywords:** epigenetics, follicular cell-derived thyroid cancer, biomarkers, inhibitors, therapy

## Abstract

The incidence of thyroid cancer has been rising in recent years. While tumorigenesis has traditionally been attributed to the accumulation of genetic mutations in oncogenes and tumor suppressor genes, increasing attention has been directed toward the role of epigenetic regulation in cancer development. Since the 1980s, however, it has been acknowledged that the role of another key regulatory system in carcinogenesis: epigenetics, shedding light on the regulation of gene expression without altering the DNA sequence.This review synthesizes current literature on epigenetic alterations in follicular cell-derived thyroid cancers, focusing on DNA methylation, histone modifications, chromatin remodeling, and RNA regulation. Evidence indicates that dysregulation of these epigenetic processes is prevalent in thyroid cancer, influencing tumor initiation, progression, and resistance to therapy. Several epigenetic inhibitors are under development, some demonstrating synergy with existing chemotherapies and immunotherapies. Understanding these mechanisms may facilitate the development of novel, more effective strategies for early detection and treatment.

## 1 Introduction

Classical genetics has posited that genetic information is primarily determined by the DNA sequence (1). However, as scientific exploration advanced, it became evident that classical genetics alone could not fully explain certain biological phenomena. For instance, Morgan et al. demonstrated that the expression of the white gene in fruit flies is influenced by its location in either heterochromatin or euchromatin, a phenomenon linked to the local environment of the nucleus (2). Building on such findings, developmental biologist Waddington introduced the term “epigenetics” to describe the interaction between genes and environmental factors in shaping phenotypes, establishing it as a distinct field in biology (3). Epigenetics now refers to heritable modifications in gene expression that do not involve changes to the DNA sequence itself (4). These modifications can be stably transmitted through cell division, and serve as a crucial link between environmental cues and gene expression. They are essential for various biological processes including development, cell proliferation, aging and others. On the other hand, the dysregulation of epigenetic mechanisms is fundamentally involved in numerous pathophysiological processes like cancer, psychiatric diseases, cardiovascular diseases, metabolic diseases, autoimmune diseases, and so on (5–7). In the 1980s, Feinberg and Vogelstein first correlated epigenetic changes with cancer, marking a turning point in understanding cancer biology. Emerging evidence has highlighted the prevalence of epigenetic modifications in most tumors, underscoring their critical role in tumor initiation and progression (8). For instance, recent research from the Van Andel Institute revealed that the loss of two key protective proteins induces abnormal DNA methylation, converting healthy lung cells into cancer cells (9). This finding demonstrated that cancer cells can be exclusively engendered by epigenetic modifications, highlighting the significance of epigenetic changes in cancer. Thus, understanding the underlying epigenetic mechanisms driving cancer occurrence and progression is imperative.

Thyroid cancer stands as the most common malignant tumor of the endocrine system, with a global incidence that continues to rise (10, 11). It was the ninth most common cancer worldwide in 2020 and is projected to become the fourth most diagnosed cancer in the United States by 2030, imposing a substantial socioeconomic burden (12). Follicular cell-derived thyroid cancers include papillary thyroid cancer (PTC), follicular thyroid cancer (FTC), and anaplastic thyroid cancer (ATC). Differentiated thyroid cancer (DTC), which include PTC and FTC, account for over 90% of cases and typically have an excellent prognosis, with a 5-year survival rate exceeding 90% (13, 14). ATC is a rare form of thyroid cancer, comprising less than 5% of all thyroid cancers, but displays a highly aggressive phenotype (15, 16). ATC has a dismal prognosis and high lethality and accounts for 14–39% of thyroid cancer related death with a median survival of just 5–6 months (17). The diverse molecular, histological, and clinical profiles of these subtypes necessitate the identification of reliable biomarkers for precise diagnosis and effective management.

Early detection and accurate diagnosis are paramount for improving thyroid cancer outcomes. Clinically, the diagnosis of thyroid nodules is mainly based on physical examination by clinicians and thyroid ultrasound examination. However, differentiating between benign and malignant thyroid nodules remains challenging. Fine-needle aspiration biopsy (FNAC) provides high sensitivity (62–89%) and specificity (71–100%), approximately 15–30% of thyroid nodules yield indeterminate results (18–21). This diagnostic uncertainty complicates clinical decision-making, as benign nodules generally require observation, while malignant ones necessitate surgical intervention (22). Therefore, there is a pressing need for non-invasive diagnostic tools to differentiate benign from malignant nodules preoperatively.

Surgical resection remains the cornerstone of thyroid cancer management, with radioactive iodine (RAI) therapy serving as a standard adjuvant treatment for DTC following thyroidectomy (23). Although the prognosis for most DTC patients is favorable, approximately two-thirds of metastatic DTC cases exhibit RAI resistance, limiting therapeutic options (24). For ATC, a multidisciplinary approach combining surgery, radiotherapy, and chemotherapy is employed, yet the prognosis remains grim (25). These thyroid cancers currently account for the majority of thyroid cancer-related deaths, highlighting the necessity of exploring molecular mechanisms driving thyroid cancer progression and developing more effective therapeutic strategies.

**Figure 1:**
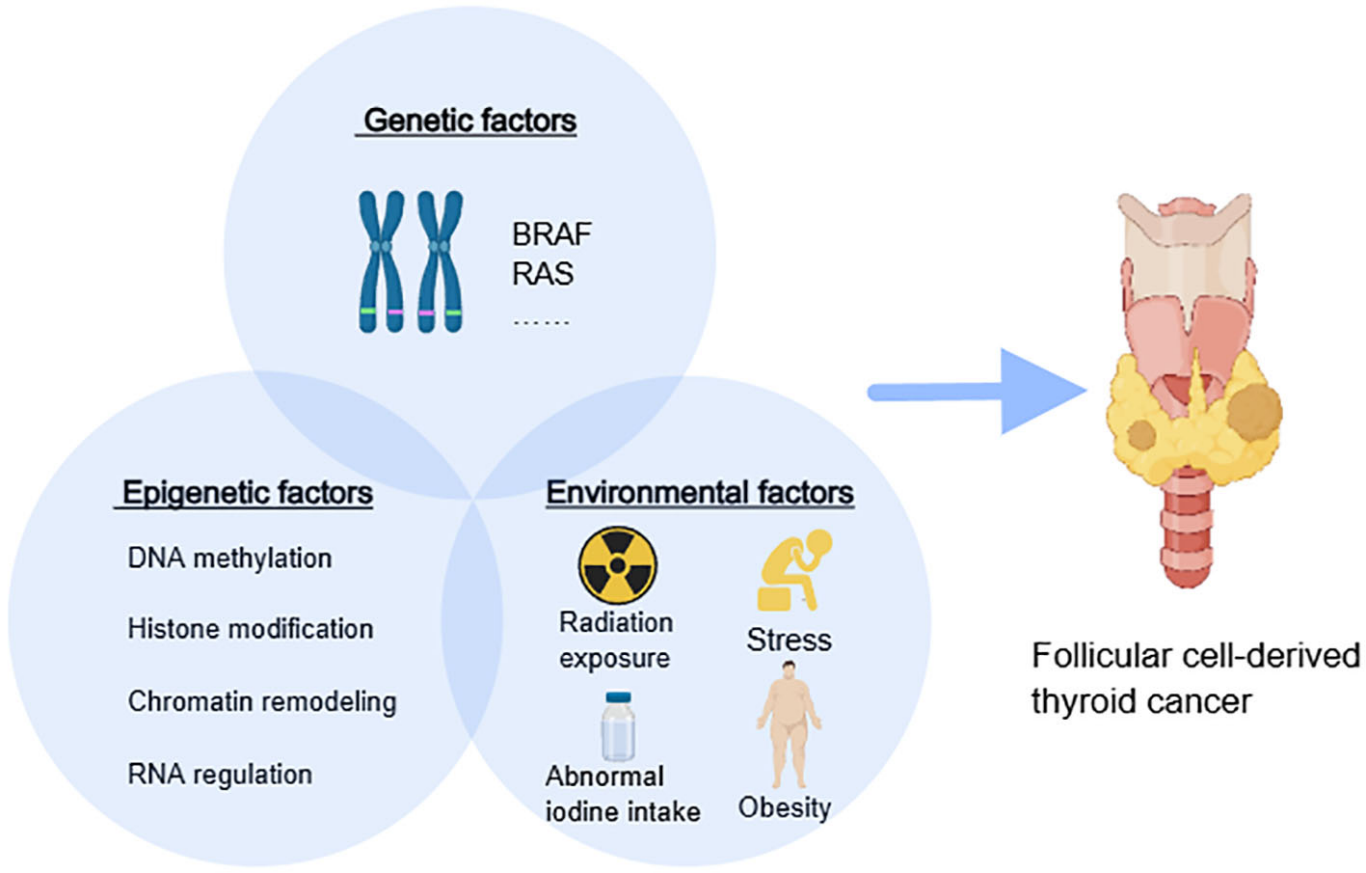
The interaction among epigenetics, genetics, and the environment. *BRAF, B-Raf proto-oncogene serine/threonine kinase*; *RAS, rat sarcoma gene family*.

Epigenetic modifications are heritable but also differ considerably based on tissue types, different developmental stages, and environmental factors (26). Epigenetics holds substantial implications for health, disease, and genetic disorders, as environmental and lifestyle factors can influence epigenetic changes, affecting an individual’s health and disease risk. While the precise etiology and pathogenesis of thyroid cancer remain incompletely understood, some risk factors for thyroid cancer have been identified, most of which can be detected and prevented early. These risk factors primarily include genetic factors (e.g., gene mutations in *B-raf proto-oncogene serine/threonine kinase (BRAF), rat sarcoma (RAS)* gene family, family history of thyroid cancer), demographic factors (e.g., higher incidence in females, white and Asian populations), and lifestyle habits (e.g., obesity, abnormal iodine intake, history of radiation exposure, stress) (27, 28). The phenotype is shaped by a complex interplay of environmental factors, genetic elements, and epigenetic mechanisms that interact with each other (**Figure 1**).

Mounting evidence indicates that epigenetic modifications play a significant role in the initiation and progression of follicular cell-derived thyroid cancers (29). However, on the one hand, current literature predominantly focuses on isolated events such as DNA methylation or histone modifications, neglecting their dynamic interplay with chromatin architecture and signaling pathways. For instance, recent findings reveal that SETMAR-mediated activation of SMARCA2, a core component of the switching defective/sucrose nonfermenting (SWI/SNF) chromatin remodeling complex, drives thyroid cancer differentiation by altering chromatin accessibility of key differentiation genes (30). On the other hand, the progress in translating epigenetic insights into precise treatments in clinical practice is lagging behind (31). Therefore, this review provides an overview of the prevailing mechanisms of epigenetic modifications in follicular cell-derived thyroid cancer including PTC, FTC ATC, prospects the future development of epigenetics and postulates opportunities for applications in thyroid diseases.

## 2 Epigenetic alterations in thyroid cancer

**Figure 2:**
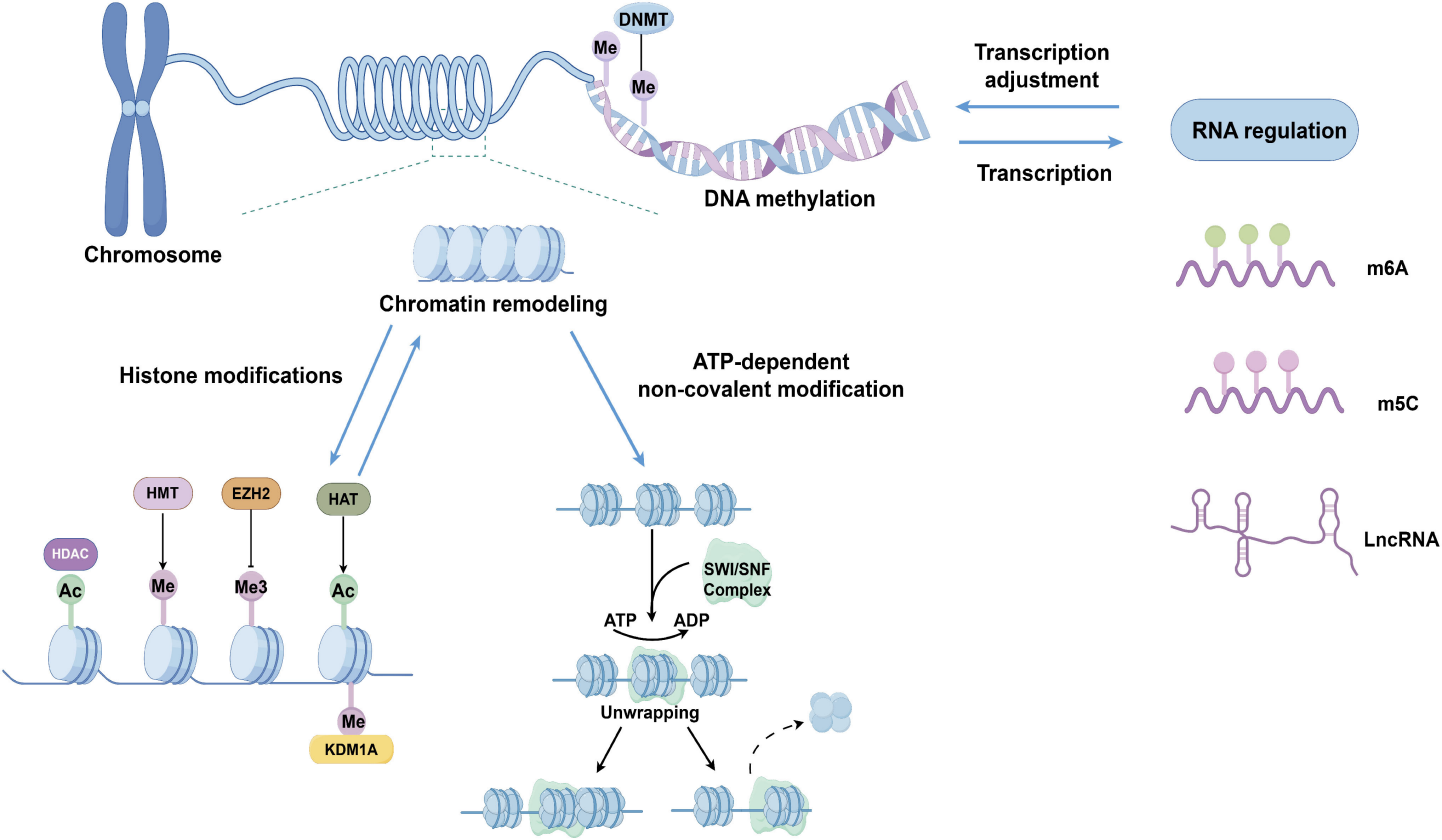
Epigenetic mechanisms involved in follicular cell-derived thyroid cancer: DNA methylation, histone modifications, chromatin remodeling, and RNA regulation. DNMT, DNA methyltransferase; Me, methylation; Me3 trimethylation Ac, acetylation; HDAC, histone deacetylase; KDM1A, lysine demethylase 1A; HMT Histone methyltransferase; EZH2, enhancer of zeste homolog 2; HAT, histone acetyltransferase; SWI/SNF, switching defective/sucrose nonfermenting; lncRNA, long non-coding RNA.

Thyroid cancer, especially follicular cell-derived thyroid cancer, has some unique epigenetic patterns, such as relatively low whole genome methylation levels, specific non-coding RNA expression patterns, and specific histone modification patterns, which are closely related to the occurrence and development of thyroid cancer (32, 33). Compared with other types of cancer, the epigenetic features of thyroid cancer may be more stable and have specific molecular markers, providing potential research directions for early diagnosis, prognosis evaluation, and targeted therapy of thyroid cancer. Epigenetic modifications primarily regulate chromatin structure and gene expression through four mechanisms, including DNA methylation, histone modifications, chromatin remodeling, and RNA regulation (**Figure 2**) (34). These aberrant epigenetic modifications are associated with all stages of tumor development, covering tumor genesis, invasion, metastasis, as well as therapy resistance (35).

### 2.1 DNA methylation

DNA methylation, one of the earliest discovered epigenetic mechanisms, plays a pivotal role in regulating diverse cellular processes, including differentiation, proliferation, chromosomal stability, genomic imprinting, and X-chromosome inactivation (36). Aberrant DNA methylation is frequently implicated in various diseases, including cancers. DNA methylation refers to the covalent addition of a methyl group to the 5-position carbon of cytosine in cytosine-phosphate-guanine (CpG) island (regions in DNA with a high density of cytosine-guanine dinucleotides), forming 5-methylcytosine (5mC) (37). This process is mediated by DNA methyltransferases (DNMTs), which play essential roles in establishing and maintaining DNA methylation patterns (38). In cancer cells, DNA methylation alterations are characterized by hypermethylation of promoter CpG islands and global genomic hypomethylation, leading to the activation of oncogenes and inactivation of tumor suppressor genes (39, 40). Promoter hypermethylation of tumor suppressor genes silences their expression, facilitating tumorigenesis. Tumor suppressor genes such as *RAS association domain family 1 isoform A*, *phosphatase and tensin homolog*, *retinoic acid receptor 2*, and *tissue inhibitor of metalloproteinase 3 (TIMP3)* exhibit hypermethylation in thyroid cancer, leading to their inactivation (41–43). For example, *TIMP3* promoter methylation in papillary thyroid cancer (PTC) correlates with tumor aggressiveness and the presence of the *BRAF^V600E^* mutation, underscoring its potential role as an oncosuppressor (44). Thyroid-specific genes such as thyrotropin-stimulating hormone receptor (*TSHR*) and sodium-iodide symporter (*NIS*) also undergo abnormal methylation in thyroid cancer. This results in reduced iodine uptake and resistance to RAI therapy (45, 46). DNA hypomethylation, one of the characteristics of tumor cells, is considered to be associated with chromosomal instability, causing aberrant cellular proliferation and ultimately inducing tumors (47). DNA hypomethylation in repetitive sequences, such as Alu elements, contributes to genomic instability and the activation of oncogenes. In healthy cells, the pericentric heterochromatin is highly methylated, which effectively silences repetitive genomic sequences, thereby ensuring the integrity and stability of the genome. However, in numerous tumor cells, this regulatory mechanism becomes disrupted, resulting in the loss of DNA methylation in regions that are typically inactive. Consequently, transposons are reactivated and can integrate into any genomic site, thereby inducing mutations and genomic instability. These alterations may potentially contribute to tumor progression or various disease stages. Alu hypomethylation has been observed in distant metastatic DTC and ATC, suggesting a role in tumor progression and dedifferentiation (48).

DNA methylation alterations often occur early in tumorigenesis, making them promising biomarkers for early cancer detection (49). Cell-free DNA (cfDNA) carrying DNA methylation information has emerged as promising non-invasive targets for cancer detection (50). Hong et al. developed and validated ThyMet classifier, a cfDNA-based methylation marker panel that non-invasively and accurately differentiates PTC from benign thyroid nodule (BTN) (51). The ThyMet demonstrated enhanced performance in differentiating PTC from BTN compared to thyroid ultrasonography grades, while maintaining a comparable sensitivity. The integration of ThyMet and thyroid ultrasonography grades further improved the accuracy. In a word, ThyMet combined ultrasonography has complementary advantages and can provide an effective way to optimize the management of thyroid nodule population. DNA hydroxymethylation, another type of DNA modification, serves as a sign of active DNA demethylation processes (52). Reduced 5-hydroxymethylcytosine (5-hmC) levels have been documented in PTC and ATC, particularly in cases with telomerase reverse transcriptase (*TERT*) promoter mutations, highlighting its potential as a biomarker for distinguishing cancer patients from healthy individuals (53, 54).

Emerging evidence also links DNA methylation to drug resistance (55, 56). Downregulation of tumor suppressor expression by DNA methylation promotes tumor growth and enhances drug tolerance. For instance, promoter hypermethylation of insulin-like growth factor binding protein-3 (*IGFBP-3*), a tumor suppressor gene, has been associated with enhanced tumor growth and drug resistance (57).

From a therapeutic perspective, DNMT inhibitors such as decitabine and 5-azacytidine, approved for treating myelodysplastic syndromes, have shown promise in thyroid cancer (**Table 1**). They are nucleoside analogues which bind to DNA and form irreversible complexes with DNMTs during the S phase of the cell cycle, leading to the degradation of DNMTs. Studies have demonstrated that decitabine treatment effectively inhibits the proliferation of both undifferentiated and differentiated thyroid cancer cells (58, 59). Notably, a phase II clinical trial revealed that patients with metastatic PTC or FTC, who were unresponsive to RAI, exhibited recovered RAI uptake in metastatic lesions subsequent to decitabine therapy. This suggests the combination of DNMT inhibitors and radioactive iodine therapy may provide a new option for cancer patients who do not respond well to current treatment options. Iodide serves as an essential constituent of thyroid hormones and is actively transported into thyroid follicular cells via the basilar membrane-bound sodium-iodide symporter (NIS) protein. The functional integrity of NIS is critical for the effective concentration of radioactive iodine within the thyroid gland, where it becomes incorporated into colloid and decomposes into cytotoxic beta and gamma rays, inducing DNA damage, apoptosis, and cell death. Consequently, the absence or dysfunction of NIS expression is postulated to play a significant role in the development of iodine-resistant thyroid tumors (60). Hypermethylation of the NIS gene promoter and global epigenetic dysregulation of differentiation related genes may lead to the development of RAI resistance and affect clinical efficacy (61).

**Table 1:** Progress in clinical trials related to HDAC and DNMT inhibitors in thyroid cancer.

NCT number	Drug type	Status	Phase	Year	Name of the trial	References
NCT03002623	HDAC Inhibitor	Terminated	II	2016-2018	CUDC-907 Treatment in People With Metastatic and Locally Advanced Thyroid Cancer	(124, 125)
NCT00134043	HDAC Inhibitor	Completed	II	2005-2009	Suberoylanilide Hydroxamic Acid in Treating Patients With Metastatic and/or Locally Advanced or Locally Recurrent Thyroid Cancer	(126)
NCT01013597	HDAC Inhibitor	Completed	II	2010-2016	Trial of LBH589 in Metastatic Thyroid Cancer	NA
NCT00048334	HDAC Inhibitor	Completed	I	2002-2010	Depsipeptide to Treat Thyroid and Other Advanced Cancers	(127–129)
NCT00098813	HDAC Inhibitor	Completed	II	2004-2009	Romidepsin in Treating Patients With Recurrent and/or Metastatic Thyroid Cancer That Has Not Responded to Radioactive Iodine	NA
NCT00413322	HDAC Inhibitor	Completed	I	2005-2008	Study of PXD101 Alone and in Combination With 5-Fluorouracil (5-FU) in Patients With Advanced Solid Tumors	NA
NCT00413075	HDAC Inhibitor	Completed	I	2006-2011	Study of Oral PXD101 in Patients With Advanced Solid Tumors or Lymphoma	NA
NCT01638533	HDAC Inhibitor	Completed	I	2012-2020	Romidepsin in Treating Patients With Lymphoma, Chronic Lymphocytic Leukemia, or Solid Tumors With Liver Dysfunction	NA
NCT00085293	DNMT Inhibitor	Completed	II	2004-2014	Decitabine in Treating Patients With Metastatic Papillary Thyroid Cancer or Follicular Thyroid Cancer Unresponsive to I-131	NA

HDAC, Histone deacetylase; DNMT, DNA methyltransferase; I, Iodine; NA, Not available (no formal publication).

DNA methylation holds significant promise in thyroid cancer pathogenesis, offering valuable diagnostic and therapeutic potential. Molecular diagnostics targeting methylation alterations can complement existing diagnostic methods, while DNMT inhibitors hold promise for overcoming treatment resistance and enhancing patient outcomes. Continued exploration of DNA methylation biomarkers and therapies will likely transform thyroid cancer management.

### 2.2 Histone modifications

Histones, the most abundant proteins bound to DNA, are key components of chromatin and play a vital role in gene regulation. Histones have five main types: H1, H2A, H2B, H3 and H4. They have similar structures, but differ slightly in some ways to accommodate different chromosomal structures and functions. Post-translational modifications to the N-terminal tails of histones, including acetylation, methylation, ubiquitination, and phosphorylation, regulate chromatin accessibility and transcriptional activity (62). Relaxed chromatin states facilitate promoter region accessibility, promoting gene expression, while condensed chromatin restricts access, silencing genes (63, 64). Among these, acetylation and methylation are the most extensively studied and are recognized as critical epigenetic markers (65).

#### 2.2.1 Histone acetylation

Histone acetylation is dynamically regulated by histone acetyltransferases (HATs) and histone deacetylases (HDACs). Histone acetylation occurs mostly on the N-terminal lysine residues of histones H3 and H4. Acetylation neutralizes the positive charge on lysine (K) residues, weakening histone-DNA interactions and resulting in an open chromatin configuration that promotes transcription (66, 67). Studies have revealed a strong correlation between histone acetylation levels and thyroid cancer progression. Pupppin et al. reported that acetylation lysine residues at positions 4 and 9 of histone H3 were elevated in follicular adenomas, PTC, FTC, and ATC compared with normal tissue, with different characteristics in different subtypes (68). Specifically, acetylation at H3K18 was observed in differentiated thyroid cancers but remained unaltered in ATC, suggesting its potential as a marker for disease progression. Further research is warranted to explore whether these histone acetylation profiles can improve the diagnostic accuracy of fine-needle aspiration cytology (FNAC) in thyroid nodules.

Histone deacetylase inhibitors (HDACis) are promising agents for reprogramming thyroid cancer cells by restoring histone acetylation (69–71). HDACis, such as valproic acid (VA), panobinostat, vorinostat (SAHA), and trichostatin A (TSA), have demonstrated the ability to reinduce *NIS* expression, thereby enhancing iodine uptake in thyroid cells (72–74). However, clinical efficacy has been limited. For instance, a phase II clinical trial using VA in patients with radioiodine-refractory follicular thyroid cancer revealed only modest reductions in serum thyroglobulin levels without significant tumor shrinkage (75). Despite these limitations, HDACis have shown potential in combination therapies. SAHA, when combined with chemotherapeutic agents such as doxorubicin, carboplatin, and paclitaxel, exhibited synergistic anti-tumor effects (76). Moreover, panobinostat combined with MAP kinase inhibitors (MAPKi) demonstrated enhanced *BRAF^V600E^*-dependent redifferentiation effects, highlighting the potential for combination strategies to improve therapeutic outcomes (72).

#### 2.2.2 Histone methylation

Histone methylation occurs primarily at arginine and lysine residues of histones H3 and H4 and is mediated by histone methyltransferases (HMTs) and histone demethylases (HDMs) (77). Histone lysine residues can be monomethylated, dimethylated or trimethylated (m1, m2 and m3, respectively) (78). Histone methylation can activate or repress gene transcription, depending on which residue is methylated. For example, methylation at H3K4 is associated with transcriptional activation, while methylation at H3K9 is linked to gene repression (79). Lysine-specific demethylase 1A (LSD1/KDM1A), the first identified histone lysine demethylase, plays a critical role in thyroid cancer progression by regulating stemness and activating the Wnt/β-catenin signaling pathway (80, 81). KDM1A demethylates H3K4me1/2 of the *adenomatous polyposis coli 2 (APC2)* promoter region and the nonhistone substrate *HIF-1α (hypoxia-inducible factor 1α)*, inhibiting *APC2* transcription and activating HIF-1α/microRNA-146a/Dickkopf-1 axis, resulting in activating Wnt signaling pathway (79). GSK-LSD1, a KDM1A inhibitor, has demonstrated significant efficacy in inhibiting thyroid cancer progression and increasing chemotherapy sensitivity, suggesting potential clinical applications for advanced thyroid cancers (79).

Enhancer of zeste homolog 2 (EZH2), a key component of polycomb repressive complex 2 (PRC2), mediates H3K27 trimethylation, repressing transcription (82). The combination of the EZH2 inhibitor tazemetostat with MAPKi has shown promising results in PTC cells harboring *BRAF^V600E^* mutations. Tazemetostat alone modestly increased iodine-metabolizing gene expression and radioiodine uptake, effects significantly enhanced when combined with MAPKi (83). This combination synergistically reduced H3K27 trimethylation and improved PTC differentiation, suggesting its potential for differentiation therapy in radioiodine-refractory thyroid cancers.

Histone modifications, particularly acetylation and methylation, are emerging as critical regulators of thyroid cancer progression. The integration of histone modification profiling into diagnostic workflows, such as FNAC, may enhance preoperative diagnostic precision. Additionally, HDACis and HMT inhibitors hold promise in therapeutic strategies (**Table 1**), especially in combination with targeted therapies. Future research should focus on translating these findings into clinical applications to improve the management and outcomes of thyroid cancer patients.

### 2.3 Chromatin remodeling

DNA wraps around histones to form nucleosomes, the fundamental repeating subunits of chromatin (84). Chromatin remodeling refers to the dynamic modification of chromatin structure, which regulates gene expression by altering DNA accessibility for transcription factors and RNA polymerases (85, 86). Chromatin remodeling exists in two forms: highly compressed and silent heterochromatin, and relatively open and active euchromatin. In euchromatin, the DNA is in an “open” state, allowing transcription factors and RNA polymerases to bind to the DNA, thus activating or promoting gene expression. On the other hand, heterochromatin will hinder this process, causing gene expression to be blocked. Chromatin remodeling involves two main mechanisms (1): chemical modifications of histones, as previously discussed, and (2) ATP-dependent chromatin remodeling driven by specialized protein complexes that use ATP hydrolysis to reposition nucleosomes and alter DNA-histone interactions (87, 88). Four primary families of chromatin remodeling complexes have been identified based on structural and functional domains: SWI/SNF, imitation SWI (ISWI), chromodomain helicase DNA binding (CHD), and inositol requiring 80 (INO80) (89).

The SWI/SNF complex, also known as the BRG1/BRM-associated (BAF) complex, uses ATP hydrolysis to remodel nucleosomes, thereby enhancing DNA accessibility for transcriptional machinery (90). The complex binds to promoters, enhancers, and DNA replication initiation sites, and interacts with proteins involved in various cellular processes like the cell cycle (91). A study by Saqcena et al. demonstrated the essential role of SWI/SNF complexes in maintaining thyroid cell differentiation and suppressing thyroid cancer progression. Loss of SWI/SNF subunits in *BRAF^V600E^*-mutant thyroid cells reduced transcription factor expression and chromatin accessibility, impairing differentiation and radioiodine uptake (92). Compared with normal thyroid cells, *BRAF^V600E^*-mutant mouse thyroid cell lines showed lower expression of transcription factors and the accessibility to specific DNA binding sites, resulting in obstacles to thyroid differentiation gene expression and impaired RAI uptake. Blocking the MAPK pathway can reverse these effects in PTC without *BRAF* mutation, but not in *BRAF*-mutant thyroid cancer due to loss of SWI/SNF subunits, rendering them insensitive to its redifferentiation effects. These findings highlight SWI/SNF complexes as pivotal for thyroid cancer differentiation and potential therapeutic targets for RAI-refractory thyroid cancers.

### 2.4 RNA regulation

#### 2.4.1 RNA modifications

It is well known that more than 170 types of RNA modifications have been discovered thus far that regulate gene expression, among which methylation modification is one of the most dominant forms (93). RNA methylation participates in the post-transcriptional gene expression regulation processes like RNA degradation, alternative, splicing and translational efficiency regulation (94).

N6-methyladenosine (m6A) methylation refers to the addition of a methyl group to the 6th nitrogen atom of the adenine base in RNA, facilitated by methyltransferases (95). m6A regulates RNA stability, splicing, translation, and degradation. Its role in cancer progression is context-dependent, with methyltransferase-like protein 3 (METTL3), a core methyltransferase, exerting both oncogenic and tumor-suppressive effects in cancers (96). METTL3 stabilizes the mRNA of six-transmembrane epithelial antigen of prostate 2 (STEAP2) through m6A modification, which is recognized by the m6A reader protein YTH N6-methyladenosine RNA-binding protein F1 (YTHDF1). STEAP2 acts as a tumor suppressor by inhibiting the Hedgehog signaling pathway and epithelial-to-mesenchymal transition (EMT), thereby reducing PTC cell proliferation, invasion, and migration. Rescue experiments further demonstrated that silencing STEAP2 reverses the tumor-suppressive effects induced by METTL3 overexpression, highlighting the METTL3-STEAP2 axis as a therapeutic target in PTC (97). METTL3 also plays a crucial role in maintaining cell viability and proliferation in ATC. Silencing METTL3 reduces overall m6A levels and significantly inhibits cell viability in ATC cell lines, demonstrating its importance in this aggressive thyroid cancer subtype (98). High-throughput sequencing revealed METTL3-dependent m6A modification on mRNAs associated with key pathways such as apoptosis and angiogenesis, suggesting potential pathways for therapeutic intervention. METTL3 promotes the stability of the long non-coding RNA LINC00894 through m6A modification in a YTHDC2-dependent manner. LINC00894 exerts a tumor-suppressive effect in PTC by inhibiting lymphangiogenesis and cancer cell proliferation via the Hippo signaling pathway (99). Downregulation of LINC00894 is associated with PTC progression, lymph node metastasis, and poor patient prognosis.

5-methylcytosine (m5C) modification refers to the addition of an active methyl group from the donor to the 5th carbon of the cytosine base in RNA (100). The RNA methyltransferase NOP2/Sun RNA Methyltransferase 2 (NSUN2) is significantly upregulated in ATC and promotes codon-dependent oncogenic translation by stabilizing tRNA (101). The study reveals that NSUN2 is significantly upregulated in ATC and is associated with tumor dedifferentiation and poor prognosis. NSUN2 enhances the stability of tRNA by catalyzing tRNA m5C modification, thereby supporting selective codon-dependent oncogenic protein synthesis. NSUN2 also promotes the maturation of its upstream transcription factor c-Myc and exhibits broad anti-cancer effects in both *in vitro* and *in vivo* experiments. Moreover, NSUN2 maintains the drug resistance of cancer cells to chemotherapy drugs. The research results indicate that NSUN2-mediated m5C tRNA modification is closely related to the progression of ATC, providing a new molecular basis for the development of potential therapeutic strategies targeting ATC.

m6A and m5C RNA modifications play crucial roles in thyroid cancer by regulating gene expression and translation. m6A, driven by METTL3, modulates key oncogenic and tumor-suppressor pathways, while m5C, mediated by NSUN2, supports codon-dependent translation for cancer progression. Both modifications show potential as biomarkers and therapeutic targets, offering new strategies for diagnosis, prognosis, and treatment of thyroid cancer (102, 103).

#### 2.4.2 Non-coding RNAs

Only a small fraction of the transcriptional products of the human genome are translated into proteins to participate in biological activities, while the majority are non-coding RNAs (ncRNAs), constituting more than 90% of the genome. Recent studies have shown that ncRNAs, especially long non-coding RNAs (lncRNAs), play an important role in the occurrence and progression of thyroid cancer (104).

LncRNAs, longer than 200 nucleotides, can be located in the nucleus or cytoplasm and serve as key regulators of tumor pathogenesis, playing critical roles in tumor cell invasion, apoptosis and metastasis (105–107). LncRNAs can mediate the recruitment of chromatin-regulatory complexes to specific genomic loci and regulate gene expression through chromatin modifications (108–110). Common lncRNAs implicated in thyroid cancer include *HOX antisense intergenic RNA (HOTAIR)*, *papillary thyroid carcinoma susceptibility candidate 3*, *plasmacytoma variant translocation 1*, *metastasis associated lung adenocarcinoma transcript 1*, *growth arrest* sp*ecific 5 (GAS5)* and *BRAF-activated non-protein coding RNA* (111–114). Up-regulation of *HOTAIR* has been found in thyroid cancer and is associated with metastasis and poor prognosis (114). Thyroid cancer cells exhibit upregulated expression of *HOTAIR* compared to normal thyroid cells. Additionally, knockdown of *HOTAIR* inhibited thyroid cancer cell growth and invasion. Thus, lncRNAs hold promise as noninvasive and readily accessible biomarkers for the diagnosis and prognosis of thyroid cancer. In addition, lncRNAs in the cytoplasm are involved in the regulation of mRNA stability and function as miRNA sponges, thereby regulating gene expression (115). A study conducted by Li et al. found that *GAS5* acts as a sponge for miR-362-5p, promoting sensitivity of thyroid cancer cells to iodine 131 by upregulating *suppressor of morphogenesis in genitalia 1 (SMG1)* and deactivating the Akt/mammalian target of rapamycin signaling pathway (112). The overexpression of *GAS5* enhanced sensitivity to iodine 131 and inhibited the growth of thyroid cancer cells, while the upregulation of miR-362-5p had the opposite effect. The upregulation of miR-362-5p counteracted the effects of *GAS5*, and the overexpression of *SMG1* negated the impact of miR-362-5p upregulation on iodine 131-resistant thyroid cancer cells. *GAS5* competitively binds to miR-362-5p, and *SMG1* is targeted by miR-362-5p.

Well-known functional ncRNAs are basically involved in epigenetic modification. They mainly function on the mRNA of target genes, and down-regulate the expression of target genes by degrading mRNA or inhibiting their translation. As research on ncRNAs in thyroid cancer progresses, lncRNAs are poised to offer significant potential for application in the diagnosis and prognosis assessment of thyroid cancer. LncRNAs are associated with treatment efficacy and prognosis, influencing radiotherapy resistance/sensitivity. Although there are currently no drugs targeting for the treatment of thyroid cancer that target non-coding RNAs, non-coding RNAs appear to be promising therapeutic targets that merit further research. Nevertheless, further investigation is required to elucidate the underlying molecular mechanisms and address challenges related to the translation of these findings into clinical practice.

Epigenetic mechanisms, including DNA methylation, histone modifications, chromatin remodeling, and RNA regulation, are interconnected in regulating gene expression during tumorigenesis. For instance, the methylation state of histone proteins in chromatin changes with different DNA modification modes and sequence changes, and the methylation state of histone lysine in chromatin may affect the modification of DNA itself (116, 117). These interconnected pathways shape tumor progression, metastasis, and therapeutic responses.

## 3 Future perspectives

In this review, we discuss epigenetic modifications that play a pivotal role in thyroid cancer. Firstly, we briefly introduced the concept and the characteristics of epigenetics. Then, we reviewed the mechanisms of epigenetic modifications, encompassing DNA methylation, histone modifications, chromatin remodeling and RNA regulation, and their roles in thyroid cancer. From a clinical point of view, most of the early clinical manifestations of thyroid cancer are thyroid nodules. Therefore, early detection and identification of patients with malignant thyroid nodules are an important preventive measure to reduce the risk of thyroid cancer death. The current study found that in the earliest stage of tumor development, the epigenetic changes may precede the gene mutations, thus epigenome detection is of great significance for the early diagnosis of tumors. DNA methylation and ncRNAs are promising biomarkers of thyroid cancer. Many candidate biomarkers have been identified in thyroid cancer and many epigenetic markers are under investigation (**Table 2**). The ideal biomarker set should be able to distinguish malignant and benign thyroid lesions with high diagnostic accuracy, and future studies should focus on identifying combinations of biomarkers to assist in the diagnosis of uncertain cytological cases. Different from genetic mutations, epigenetic modifications are mostly reversible. Therefore, from a theoretical point of view, it is easier to target epigenetics than genetics in cancer treatment, opening up new possibilities for cancer treatment development (118). Researchers are developing a variety of small molecule epigenetic inhibitors for tumor treatment. However, it has been found through available data that only a very small number of patients have a complete recovery from thyroid cancer (119, 120), which may be related to the significant differences in epigenetic abnormalities among different subtypes of thyroid cancer and the abnormal activation of the immune system caused by epigenetic drugs. In addition, it is worth noting that iodine levels or radiation exposure can greatly affect epigenetic changes and weaken drug sensitivity, which may be related to DNA methylation abnormalities and histone modification dysregulation caused by DNA damage and free radical generation (121, 122)

**Table 2:** Progress in clinical trials related to combination therapy strategies for follicular cell-derived thyroid cancer.

NCT number	Status	Phase	Year	Name of the trial	Therapy	References
NCT01182285	Completed	II	2010-2016	A Phase II Trial of Valproic Acid in Patients With Advanced Thyroid Cancers of Follicular Cell Origin	Valproic Acid+Liothyronine Sodium	(75)
NCT00537095	Completed	II	2007-2021	Efficacy and Safety of Vandetanib (ZD6474) in Patients With Metastatic Papillary or Follicular Thyroid Cancer	Vandetanib+Placebo	(130)
NCT04462471	Completed	I	2020-2023	Vemurafenib Plus Copanlisib in Radioiodine-Refractory (RAIR) Thyroid Cancer	I-124 PET/CT lesion dosimetry+Vemurafenib+Copanlisib	NA
NCT00668811	Completed	II	2008-2015	Sutent Adjunctive Treatment of Differentiated Thyroid Cancer (IIT Sutent)	SU011248+Sutent	(131, 132)
NCT00984282	Completed	III	2009-2017	Nexavar^®^ Versus Placebo in Locally Advanced/Metastatic RAI-Refractory Differentiated Thyroid Cancer	Sorafenib (Nexavar, BAY43-9006)+Placebo	(133–136)
NCT01723202	Unknown status	II	2012-2022	Dabrafenib With or Without Trametinib in Treating Patients With Recurrent Thyroid Cancer	Dabrafenib+Trametinib+Correlative Studies	(137, 138)
NCT04544111	Active, not recruiting	II	2020-2025	PDR001 Combination Therapy for Radioiodine-Refractory Thyroid Cancer	Trametinib+Dabrafenib+PDR001	NA
NCT06440850	Recruiting	II	2024-2026	Vemurafenib and Cobimetinib for the Treatment of Patients With High Risk Differentiated Thyroid Carcinoma With BRAFV600E Mutation	Cobimetinib+I-131+Vemurafenib	NA
NCT05668962	Recruiting	II	2023-2026	Restor. I-131 Upt. + Selpercatinib in RET F-P RAI-R TC	Selpercatinib+Sodium I-131+rhTSH	NA
NCT03506048	Terminated	II	2019-2021	Lenvatinib and Iodine Therapy in Treating Patients With Radioactive Iodine-Sensitive Differentiated Thyroid Cancer	Lenvatinib+Radioactive Iodine Therapy	NA
NCT02973997	Completed	II	2018-2023	Lenvatinib and Pembrolizumab in Differentiated Thyroid Cancers (DTC)	Lenvatinib; Lenvatinib Mesylate; Pembrolizumab	(139)
NCT03630120	Terminated	II	2018-2019	Adaptive Tyrosine Kinase Inhibitor (TKI) Therapy In Patients With Thyroid Cancer	Lenvatinib; Sorafenib; Cabozantinib; Vandetanib	NA
NCT00381641	Completed	II	2006-2024	Sunitinib Malate in Treating Patients With Thyroid Cancer That Did Not Respond to I-131 and Cannot Be Removed by Surgery	Sunitinib; Sunitinib Malate	NA
NCT00786552	Unknown status	II	2008-2013	Pemetrexed + Paclitaxel in Patients With Recurrent/Advanced Thyroid Cancer (Panthera)	Pemetrexed + Paclitaxel	NA
NCT00085293	Completed	II	2004-2014	Decitabine in Treating Patients With Metastatic Papillary Thyroid Cancer or Follicular Thyroid Cancer Unresponsive to I-131	Decitabine+I-131+Recombinant thyrotropin alfa+ F-18	NA
NCT03914300	Active, not recruiting	II	2020-2025	Testing the Combination of Cabozantinib, Nivolumab, and Ipilimumab (CaboNivoIpi) for Advanced Differentiated Thyroid Cance	Cabozantinib S-malate+Ipilimumab+Nivolumab	NA
NCT00095693	Terminated	II	2004-2011	Sorafenib Tosylate in Treating Patients With Locally Advanced, Metastatic, or Locally Recurrent Thyroid Cancer	Sorafenib tosylate+ F-18	NA
NCT00729157	Completed	II	2008-2012	Aflibercept in Treating Patients With Recurrent and/or Metastatic Thyroid Cancer That Did Not Respond to Radioactive Iodine Therapy	F-18+Ziv-Aflibercept	NA
NCT03753919	Terminated	II	2019-2024	Durvalumab Plus Tremelimumab for the Treatment of Patients with Progressive, Refractory Advanced Thyroid Carcinoma -The DUTHY Trial (DUTHY)	Durvalumab+Tremelimumab	NA
NCT01100619	Completed	I	2010-2012	A Drug-Drug Interaction Study of the Effects of XL184 (Cabozantinib) on Rosiglitazone in Subjects With Solid Tumors	Rosiglitazone+XL184	(140)
NCT01208051	Completed	I/II	2010-2020	Cediranib Maleate With or Without Lenalidomide for the Treatment of Thyroid Cancer	Cediranib+Cediranib Maleate+Lenalidomide	NA
NCT01025453	Completed	II	2009-2018	Combination of Temsirolimus and Sorafenib in the Treatment of Radioactive Iodine Refractory Thyroid Cancer	Temsirolimus+Sorafenib	NA
NCT03690388	Active, not recruiting	III	2018-2024	A Study of Cabozantinib Compared With Placebo in Subjects With Radioiodine-refractory Differentiated Thyroid Cancer Who Have Progressed After Prior Vascular Endothelial Growth Factor Receptor (VEGFR) -Targeted Therapy	Cabozantinib+Placebo	(141)
NCT00004062	Completed	I	1999-2006	Azacitidine to Restore Thyroid Function in Patients With Persistent or Metastatic Thyroid Cancer	Azacitidine+Liothyronine sodium+I-131	NA
NCT00062348	Completed	I	2003-2007	Boronophenylalanine-Fructose Complex (BPA-F) and/or Sodium Borocaptate (BSH) Followed By Surgery in Treating Patients With Thyroid Cancer, Head and Neck Cancer, or Liver Metastases	Boronophenylalanine-fructose complex+Sodium borocaptate+Conventional surgery	(142–146)
NCT00002608	Completed	II	1994-2005	Combination Chemotherapy and Tamoxifen in Treating Patients With Solid Tumors	Cisplatin+Doxorubicin hydrochloride+Tamoxifen citrate+Conventional surgery+Radiation therapy	NA
NCT00005842	Completed	I	2000-2004	Trastuzumab Plus R115777 in Treating Patients With Advanced or Metastatic Cancer	Trastuzumab+Tipifarnib	NA

I, Iodine; F, Fludeoxyglucose; rhTSH, Recombinant human thyroid-stimulating hormone; PET/CT, Positron emission tomography/computed tomography; RAI, Radioactive iodine; NA, Not available (no formal publication).

There is still much work to be done to determine the real effectiveness of epigenetic drugs alone for the treatment of thyroid cancer. Although there are many problems to be solved, epigenetic therapy has shown clear new advantages over classical genetic theory. The core point is that epigenetic therapy is used to reprogram tumor cells, rather than induce cytotoxicity, which is a completely different mechanism from chemotherapy targeted therapy. Resistance to traditional anti-tumor drugs has always been a difficult problem to solve, but epigenetic drugs can inhibit the generation of resistance, enhance the efficacy, and even directly act on the tumor cells that have developed resistance. Consequently, the concurrent administration of two or more epigenetic drugs may represent a promising strategy for enhancing the therapeutic effectiveness against tumors. For instance, HDACis TSA or VA in combination with demethylating agent 5-azacytidine inhibit cell growth in FTC and PTC cell lines (123). In addition, epigenetic therapy in combination with other therapies may significantly enhance treatment response rates in thyroid cancer, including chemotherapy, targeted drugs, and immunotherapy. Future trials should prioritize combined therapies to achieve more effective results in the treatment of thyroid cancer. In the last, more studies linking environmental exposure to epigenetic and genetic changes in thyroid cancer are needed. In summary, despite the clear evidence of epigenetic modifications in thyroid cancer, further research is needed to better understand the pathogenesis of thyroid cancer, thereby paving the way for the optimization and improvement of clinical diagnosis and treatment of thyroid cancer.
